# Design and Implementation of an Ultrasonic Flowmeter Based on the Cross-Correlation Method

**DOI:** 10.3390/s22197470

**Published:** 2022-10-02

**Authors:** Rui Ren, Hongliang Wang, Xiaolei Sun, He Quan

**Affiliations:** National Key Laboratory for Electronic Measurement Technology, Key Laboratory of Instrumentation Science and Dynamic Measurement, Ministry of Education, North University of China, Taiyuan 030051, China

**Keywords:** ultrasonic flowmeter, cross-correlation method, finite element simulation, ultrasonic time of flight

## Abstract

Ultrasonic flowmeters play an important role in industrial production, aerospace and other fields. In this paper, a high-precision ultrasonic flowmeter based on the cross-correlation method is designed, and the commercial finite element software COMSOL Multiphysics 5.6 is used to simulate the propagation process of ultrasonic waves during flow measurement, and the implementation process of the cross-correlation algorithm is simulated by Python language. The flowmeter adopts the cross-correlation algorithm to improve the measurement accuracy of ultrasonic time of flight and adopts the method of combining FPGA and an embedded microprocessor to improve operation efficiency. In order to verify the performance of the flowmeter, we tested the flowmeter on the National Institute of Metrology and the self-built test platform, using the still water dragging method, the dynamic volume method and the field comparison method, respectively. The results show that the flowmeter has the ability to test the flow under the condition of high flow velocity (26 m/s) and a pipe diameter in the range of DN6~DN1600, that the absolute value of the relative indication error does not exceed 0.815% and that the repeatability does not exceed 0.150%. The designed ultrasonic flowmeter has high measurement accuracy, good repeatability, strong stability and a wide application range.

## 1. Introduction

In the process of control and detection of industrial production, flow is one of the important reference indicators, which plays a pivotal role in the stable operation of industrial production [1]. At present, flow measurement has been widely used in medical, petroleum, gas, heating, aerospace and other fields. How to accurately measure liquid flow has become one of the issues that people need to pay close attention to in the process of production and life [2,3,4,5]. Liquid-measuring instruments mainly include electromagnetic flowmeters [6], turbine flowmeters [7], vortex flowmeters [8] and ultrasonic flowmeters [9]. Compared with other types of flowmeters, ultrasonic flowmeters have the advantages of easy installation, a simple working principle, strong adaptability to pipe diameter, high measurement accuracy and fast response speed. They have extremely important application value in various fields of military and civilian use [10,11].

The measurement principles of ultrasonic flowmeters include the frequency difference method [12], quartz oscillator method [13], beam shift method [14], time difference method [15] and so on. When the ultrasonic wave propagates in the fluid, the bubbles and fine particles contained in the fluid cause scattering and refraction of the ultrasonic signal, thus forming a frequency difference between the ultrasonic transmitting point and the receiving point. The fluid flow rate can be calculated by measuring the frequency difference. This method measures the fluid velocity according to the Doppler frequency shift of the sound wave generated by the movement of solid particles in the fluid, and requires the fluid to contain particles or bubbles, so it is not suitable for measuring pure water [16]. Mechanical effects on the crystal or electrical effects on its substitute electrical model can trigger frequency changes in the oscillator. Therefore, a physical quantity near resonance can be detected by a small change in capacitance or inductance. The quartz oscillator method is detected by the oscillator connected in series or in parallel with the piezoelectric crystal and the reactance. This method has strong sensitivity and good temperature compensation [13]. The beam deflection method realizes the measurement of flow through the angle of ultrasonic deflection, and its accuracy is low under the condition of low flow velocity, so it is suitable for the environment of high flow velocity [17]. The time difference method is to measure the fluid velocity based on the time of flight (TOF) of the ultrasonic signal along the downstream and upstream directions within a certain distance. This method has been widely used due to its simple principle and strong practicability [18].

During measurement, the TOF ultrasonic flowmeter can use the insert type to place the sensor inside the pipeline or use the outer clamp type to place the sensor outside the pipeline. In contrast, the outer clamp-type ultrasonic flowmeter does not need to destroy the pipeline. In addition, the sensor is detachable, convenient and quick to use, and has a wide range of applications, but the accuracy needs to be further improved [19]. The key to improving the measurement accuracy of the ultrasonic flowmeter is the accurate measurement of TOF. Currently, two common methods for measuring TOF are the zero-crossing detection method and the cross-correlation method [20]. Compared with the zero-crossing detection method, the cross-correlation method is only related to the similarity of the signals and is not easily interfered by the noise signal. It can more accurately determine the TOF of the ultrasonic signal along the upstream and downstream directions and improve the measurement accuracy [21].

In recent years, many researchers have made remarkable achievements in improving the measurement accuracy and stability of ultrasonic flowmeters. Kang, L. et al., University of Warwick, UK, proposed a spatial averaging method based on curved ultrasonic array transducer technology to improve the measurement accuracy and reduce the error of the measurement results, and developed a new two-dimensional curved ultrasonic array transducer to verify the measurement method [22]. Maaß, S. et al. of Leipzig University in Germany proposed a method for sub-sampling time delay difference estimation for two narrow-band ultrasonic signals, extending the application range of external clamp ultrasonic flowmeters below 50 mm/s [23]. The University of Technology Sydney, Australia, proposed a method that uses a capacitance-based liquid level sensor to determine the propagation path, combined with the fluid velocity determined by an ultrasonic sensor, to estimate the flow more accurately [24]. At the Iran University of Science and Technology, Alaeddin, M.A. et al. studied the influence of sensor position on flow measurement accuracy and proposed the best installation scheme of ultrasonic flowmeter sensor for single-channel and multi-channel flowmeters [25]. Massaad, J. et al. of Delft University of Technology in the Netherlands designed a clamp-on ultrasonic flowmeter composed of two matrix array ultrasonic transducers, which solved the problem of manually finding the horizontal distance of the ultrasonic transducer in the process of ultrasonic transducer positioning [26]. These research results have greatly promoted the widespread application of ultrasonic flowmeters.

In this work, based on the theoretical research of ultrasonic flow measurement, an ultrasonic flowmeter based on the cross-correlation method is designed, and the function verification and performance tests of the flowmeter are carried out. The rest of the paper is structured as follows. Section 2 describes the measurement principle of an ultrasonic flowmeter. Section 3 describes the modeling and simulation of ultrasonic flow measurement simulation through COMSOL software and simulates the implementation process of cross-correlation algorithm through Python. Section 4 describes the hardware and software design of the ultrasonic meter. Section 5 describes the methodology and data analysis used for the test. Section 6 summarizes the conclusions.

## 2. Measurement Principle of the Flowmeter

**Figure 1:**
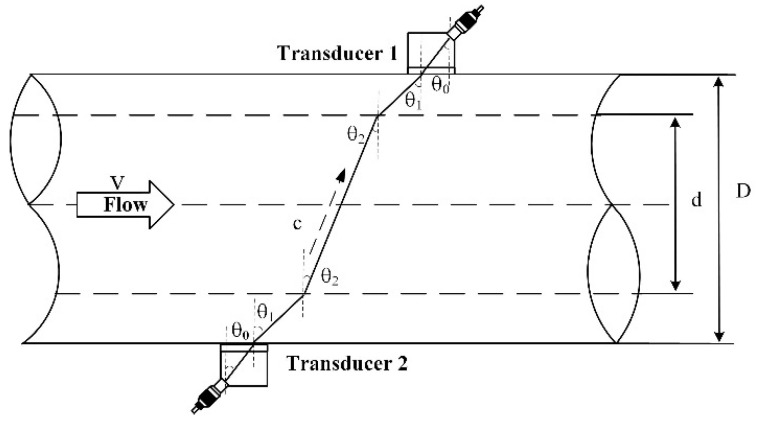
Schematic diagram of time difference measurement.

The clip-on ultrasonic flowmeter based on the cross-correlation method is improved on the basis of the time difference method. The time difference method measures the flight time difference between the upstream and downstream ultrasonic waves in the fluid within a fixed distance. According to the propagation speed of the ultrasonic wave in the fluid medium, the flow velocity of the fluid medium can be calculated, and then the flow through the pipeline can be calculated according to the diameter of the pipeline. As shown in Figure 1, the flowmeter consists of a pair of ultrasonic sensors installed on the outer surface of the pipeline. The two sensors are, respectively, clamped to the upstream and downstream of the measured pipeline at a certain angle [27]. When the ultrasonic component of the fluid flow direction is the same in the flow direction of the fluid in the measured pipeline, the transmission time between the two ultrasonic transducers is reduced. When the ultrasonic component of the fluid flow direction is opposite to the flow direction of the fluid in the measured pipeline, the transit time between the two ultrasonic transducers increases [28].

The angle between the ultrasonic transducer and the pipe is $\theta_{0}$, the angle of ultrasonic refraction from the transducer to the pipe wall is $\theta_{1}$, the angle from the pipe wall to the fluid is $\theta_{2}$, the flow velocity of the fluid is v, the propagation velocity of ultrasonic waves in the transducer is $c_{0}$, the propagation velocity in the pipe is $c_{1}$, the propagation velocity in the fluid is $c_{2}$, the inner diameter of the pipe is d and the outer diameter is D.

The relationship between the incident wave and reflected wave of ultrasonic waves in different media conforms to Snell’s law in optics [29]. The refraction relationship from the transducer to the tube wall is:

(1) $$\frac{sin\theta_{0}}{c_{0}}=\frac{sin\theta_{1}}{c_{1}},$$

The refraction relationship from the tube wall to the fluid is:

(2) $$\frac{sin\theta_{1}}{c_{1}}=\frac{sin\theta_{2}}{c_{2}},$$

where $\theta_{2}$ is the angle from the tube wall to the fluid and $c_{2}$ is the propagation speed of the ultrasonic wave in the fluid. The propagation length of the ultrasonic signal in the fluid is:

(3) $$L=\frac{d}{cos\theta_{2}},$$

In the process of ultrasonic propagation from ultrasonic transducer 2 to ultrasonic transducer 1, the downstream propagation speed is $c_{2\;}{+\;\mathrm{vcos}\theta }_{2}$, so its downstream propagation time in the fluid is [30]:

(4) $$T_{up}=\frac{L}{c_{2}+vcos\theta_{2}},$$

In the process of ultrasonic propagation from ultrasonic transducer 1 to ultrasonic transducer 2, the upstream propagation speed is $c_{2\;}{-\;\mathrm{vcos}\theta }_{2}$, so its upstream propagation time in the fluid is:

(5) $$T_{\mathrm{down}}=\frac{L}{c_{2}-vcos\theta_{2}},$$

According to Equations (4) and (5), the time difference between the upstream and downstream flight time of the ultrasonic wave can be calculated by subtracting the downstream propagation time from the upstream propagation time:

(6) $$\Delta T=T_{down}-T_{up}=\frac{2dv}{c_{2}{}^{2}-v^{2}cos^{2}\theta_{2}},$$

The time-of-flight difference can be measured by the time chip in the circuit, so the fluid flow velocity can be expressed as:

(7) $$v=\frac{-d+\sqrt{d^{2}+\Delta T^{2}c_{2}{}^{2}cos^{2}\theta_{2}}}{\Delta Tcos^{2}\theta_{2}},$$

Since the inner diameter of the pipe is determined, the instantaneous volume flow can be expressed as:

(8) $$q=\frac{\pi d^{2}}{4}v,$$

According to Equations (7) and (8), the instantaneous volume flow q can be calculated. It can be seen that the key to ensuring the measurement accuracy of the system is to accurately measure the time difference between the upstream and downstream flight time [31]. Through the cross-correlation method, the time-of-flight difference can be determined more accurately, so the measurement accuracy of the flowmeter can be improved.

## 3. Simulation and Analysis of Ultrasonic Flow Measurement

### 3.1. Background Flow in Different Fluid States

**Figure 2:**
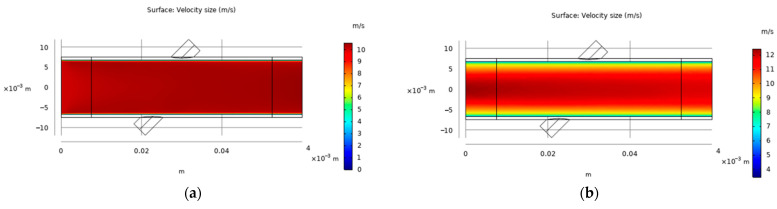
(**a**) Background average flow amplitude of laminar fluid state; (**b**) Background average flow amplitude of turbulent fluid state.

When using COMSOL software for ultrasonic flow measurement simulation, it is first necessary to simulate the fluid state in the pipeline. In this paper, the outer diameter of the pipe is 15 mm, the thickness of the pipe is 0.75 mm and the material is PVC. The average flow amplitude of the laminar background is shown in Figure 2a, and the average flow amplitude of turbulent background is shown in Figure 2b.

**Figure 3:**
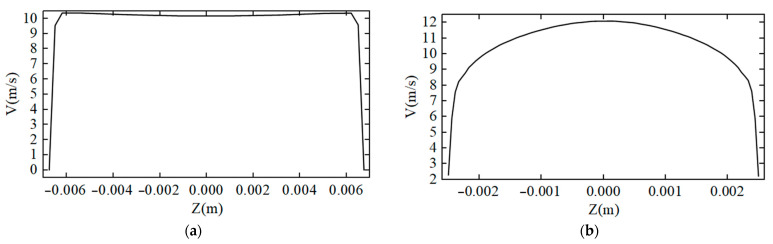
(**a**) Background velocity curve of laminar fluid state; (**b**) Background velocity curve of turbulent fluid state.

The background flow velocity curve of the laminar fluid state is shown in Figure 3a. It can be seen that the laminar flow state only exists in a transition state of flow velocity within the range of about 0.5 mm close to the pipeline, and the other positions in the pipeline are all about 10 m/s. This is because the fluid has dynamic viscosity, and the internal friction caused by the pipe to the fluid flow causes the fluid flow to lag. Figure 3b shows the background velocity curve of the turbulent fluid state. It can be seen that, in the pipeline fluid with an average velocity of 10 m/s, the fluid velocity next to the pipeline is about 4.5 m/s, and the fluid velocity in the middle of the pipeline can reach about 12.2 m/s; the fluid velocity curve in the pipeline is parabolic as a whole.

### 3.2. Influence of Pipe Diameter on Ultrasonic Transmission

Attenuation occurs during ultrasonic propagation, and the greater the distance, the weaker the signal received by the ultrasonic transducer. In this paper, the simulation analysis is carried out by taking the outer diameter of the pipe as an example of 10 mm, 15 mm and 20 mm. The angle between the transducer and the pipe is 45°, the ultrasonic emission frequency of the ultrasonic transducer is 2 MHz, the fluid in the pipe is water and the pipe material is PVC. The ultrasonic transducer on the lower side of the pipeline is a transmitter, and the ultrasonic transducer on the upper side of the pipeline is a receiver. A normal velocity is given at the transmitter as an ultrasonic transmission signal. The transmitted signal is a sine wave modulated by a Gaussian pulse, and its expression is:

(9) $$v_{n}\left(t\right){=\mathrm{Ae}}^{-\left(f_{0}{\left({t-3T}_{0}\right)}^{2}\right)}\sin\left(\omega_{0}t\right)$$

where A is the signal amplitude, $f_{0}$ is the signal frequency and t is the time variable, $\omega_{0}{=2\pi f}_{0}$, $T_{0}{=1/f}_{0}$.

**Figure 4:**
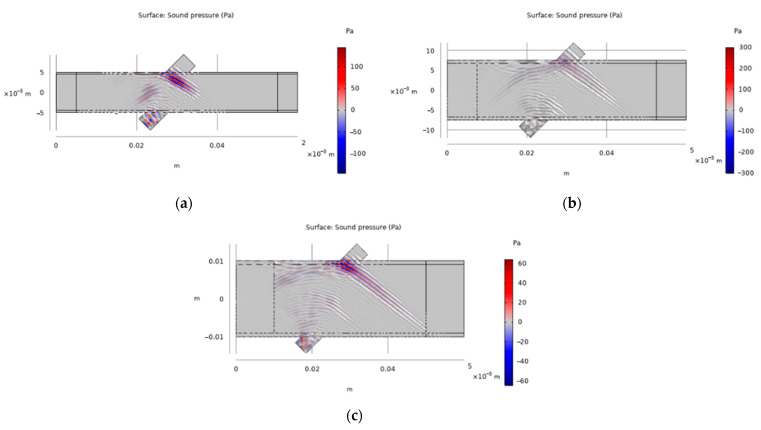
(**a**) Schematic diagram of sound pressure propagation of 10 mm pipe diameter; (**b**) Schematic diagram of sound pressure propagation of 15 mm pipe diameter; (**c**) Schematic diagram of sound pressure propagation of 20 mm pipe diameter.

Figure 4a–c are schematic diagrams of the acoustic pressure propagation simulation of the outer diameter of the 10 mm, 15 mm and 20 mm pipes, respectively. It can be seen from the figures that the ultrasonic wave transmission time from one side of the pipe to the other side under different pipe diameters is, respectively, 9.375 × 10^−6^ s, 1.3542 × 10^−5^ s and 1.6667 × 10^−5^ s. It can be seen that the larger the outer diameter of the pipe, the longer the propagation time of the ultrasonic wave from the transmitter to the receiver, and the sound pressure gradually decreases with the increase in the propagation distance of the ultrasonic wave.

**Figure 5:**
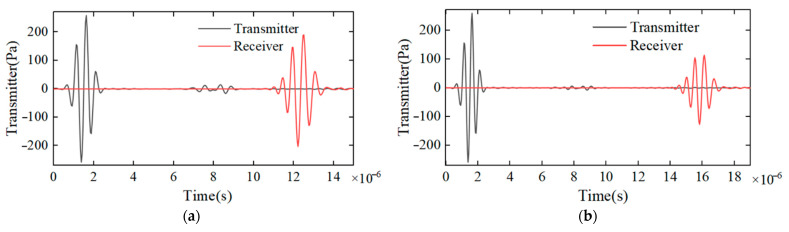

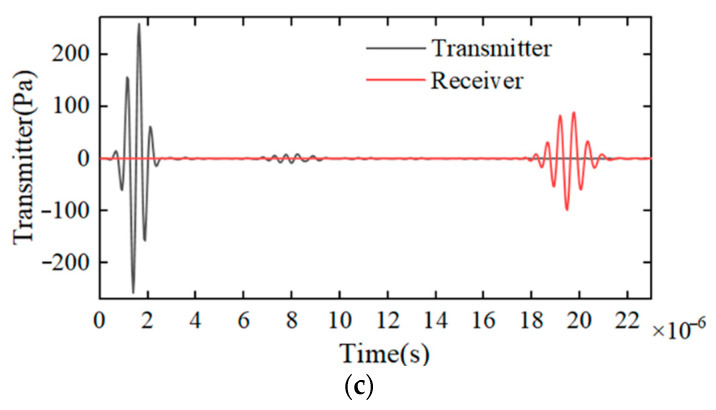
(**a**) 10 mm diameter ultrasonic transmission and reception signal; (**b**) 15 mm diameter ultrasonic transmission and reception signal; (**c**) 20 mm diameter ultrasonic transmission and reception signal.

Figure 5 shows the sound pressure waveforms of the ultrasonic transmitter and receiver under the outer diameter of the 10 mm, 15 mm and 20 mm pipes. Among them, the waveform on the left is the sound pressure at the transmitter end, the waveform on the right is the sound pressure at the receiver end and the small protrusion waveform in the middle is caused by the reflected sound pressure when the ultrasonic wave transmitted by the transmitter propagates to different media and propagates to the transmitter again. The peak sound pressure of the transmitter is about 250 Pa. The peak values of sound pressure received by the ultrasonic receiver under the outer diameter of the 10 mm, 15 mm and 20 mm pipes are about 200 Pa, 110 Pa and 90 Pa, respectively. It is also more intuitive to see that, with the increase in the outer diameter of the pipe, the ultrasonic wave propagation time becomes longer and the sound pressure becomes smaller.

### 3.3. Simulation Analysis of Ultrasonic Flow Measurement by Cross-Correlation Method

In the process of ultrasonic flow measurement, the frequency of the ultrasonic waves transmitted and received is the same in the up-current flight and down-current flight, and the signals received by the ultrasonic wave in the up-current flight and down-current flight are in the shape of a “jujube core”. Therefore, the cross-correlation algorithm can be used to measure the time-of-flight difference more accurately according to the similarity of the two received waveform shapes, thereby improving the measurement accuracy of the flowmeter.

**Figure 6:**
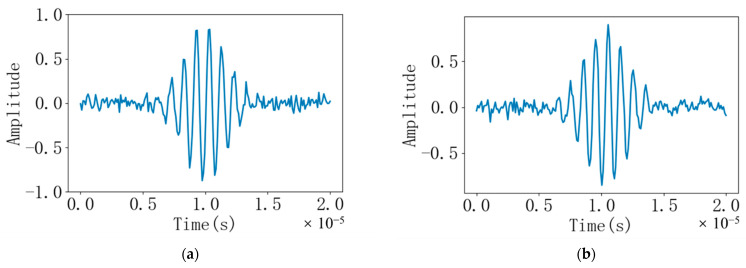
(**a**) Downstream echo signal with noise added; (**b**) Countercurrent echo signal with noise added.

Based on Python, this paper simulates the downstream echo signal and the upstream echo signal and performs cross-correlation calculation and interpolation fitting. In order to simulate the time difference between the downstream echo signal and the countercurrent echo signal, the “jujube core” waveform was sampled at a sampling rate of 10 times and 50 times the signal frequency, respectively. Then, signals with different times and zero amplitudes are inserted on both sides of the “jujube core” waveform, respectively. Finally, different noises are added to the generated echo signals with time difference, respectively. The noise is a random number with a standard deviation of 0.05 generated by Python. The two are added together to simulate the signal received by the actual receiver. The final signal is shown in Figure 6. The frequency of the signal is f = 1 MHz.

**Figure 7:**
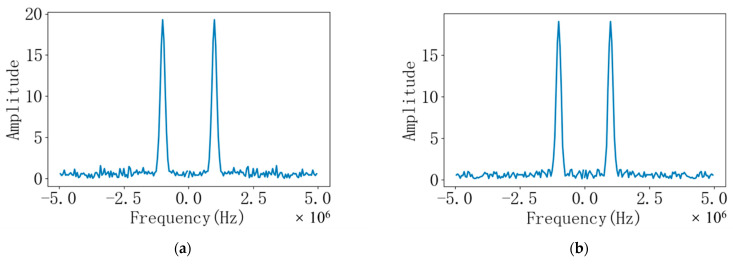
(**a**) Bilateral spectrogram of the downstream echo signal before filtering; (**b**) Bilateral spectrogram of the countercurrent echo signal before filtering.

**Figure 8:**
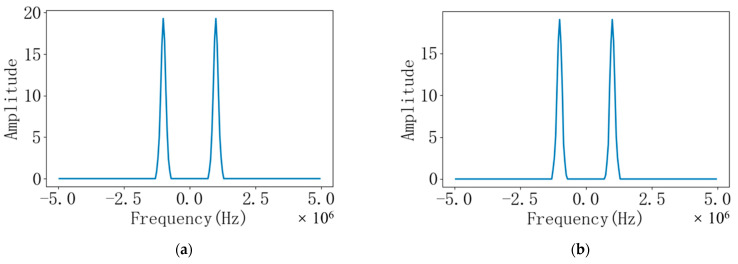
(**a**) Filtered bilateral spectrogram of downstream echo signal; (**b**) Filtered bilateral spectrogram of countercurrent echo signal.

The ultrasonic signal with noise is transferred to the frequency domain through the fast Fourier transform, which is convenient for filtering and cross-correlation calculation. Converting the discrete time domain signal shown in Figure 6 to the double-sided spectrum in the frequency domain is shown in Figure 7. It can be seen from the figure that the frequency domain signals of the downstream and upstream echoes have small amplitudes outside 0.995 MHz~1.005 MHz and −1.005 MHz~−0.995 MHz, that is, noise interference signals. As shown in Figure 8, it is a bilateral spectrogram obtained by removing the interference signal in the frequency domain of Figure 7.

**Figure 9:**
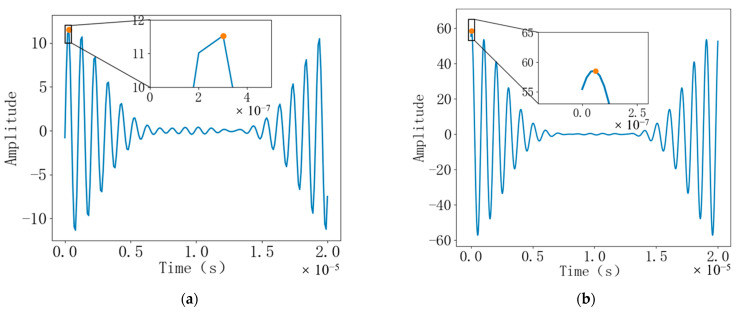
(**a**) When the sampling rate is 10 times the signal frequency, the time-domain output of the cross-correlation function; (**b**) When the sampling rate is 50 times the signal frequency, the time-domain output of the cross-correlation function.

The obtained filtered upstream echo signal in the frequency domain is conjugated and then multiplied by the downstream echo signal in the frequency domain to obtain the frequency domain output of the cross-correlation function. Then, inverse fast Fourier transform is performed on the obtained cross-correlation signal in the frequency domain to obtain the time-domain output of the cross-correlation function. The time-domain output result of the cross-correlation function is shown in Figure 9. The time corresponding to the highest point of the time-domain output is the time difference between the downstream echo signal and the upstream echo signal.

**Figure 10:**
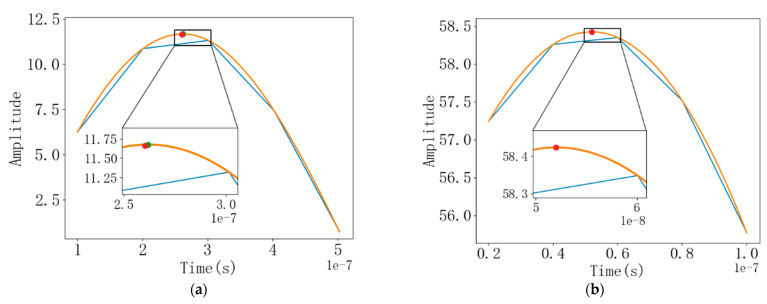
(**a**) When the sampling rate is 10 times the signal frequency, the comparison between the original function and the interpolation function; (**b**) When the sampling rate is 50 times the signal frequency, the comparison between the original function and the interpolation function.

After the cross-correlation calculation, it is necessary to use quadratic interpolation or cubic spline interpolation for calculation, in order to obtain a more accurate time difference between the downstream echo and the upstream echo and reduce the flow measurement error caused by the low ADC sampling rate in the actual use process. When the sampling rate is 10 times and 50 times the signal frequency, respectively, the interpolation calculation results based on the peak point and its left and right neighbors are shown in Figure 10. The blue broken line in the figure is a broken line graph formed by connecting the original discrete points with straight lines, and the curve is the curve after interpolation. The curve after quadratic interpolation and the orange curve after cubic spline interpolation almost coincide, and the highest point also almost coincides. The red point on the left in the figure is the highest point of cross-correlation after quadratic interpolation, and the green point on the right is the highest point of cross-correlation after cubic spline interpolation. The ordinate of the output of the cross-correlation function represents the similarity of the forward and backward echo signals corresponding to the time T. If the sampling frequency is increased, the similarity of the downstream and upstream echo signals increase, that is, the amplitude of the cross-correlation function in the figure increases. The higher the sampling frequency, the more sampling points, the higher the degree of restoration of the signal and the more accurate the measurement result, but the high sampling frequency requires a lot of hardware and the processing data are too large. In the actual application process, the appropriate sampling frequency should be selected according to the subsequent circuit design standards.

In order to further compare the influence of different interpolation fittings on the measurement error, the theoretical time difference between the downstream echo and the upstream echo is gradually increased in units of 10 ns, and then the error is calculated without interpolation, by quadratic interpolation and by cubic spline interpolation. The calculation results are shown in Table 1.

After the analysis of Table 1, it can be seen that, when the theoretical time difference between the downstream echo and the upstream echo is gradually increased in units of 10 ns, the actual time difference without interpolation is 2.0 × 10^−7^ s and the actual average error without interpolation is 12.2581%. The average error after sub-interpolation is 0.9068% and the average error after cubic spline interpolation is 0.9131%. Therefore, it can be known that, when the time difference between the downstream echo and the upstream echo gradually increases in units of 10 ns, the use of quadratic interpolation can reduce the system error, thereby improving the system measurement accuracy. In addition, the average error of both quadratic interpolation and cubic spline interpolation system is within 1%.

## 4. Design of Ultrasonic Flowmeter

### 4.1. Overall Scheme Design

**Figure 11:**
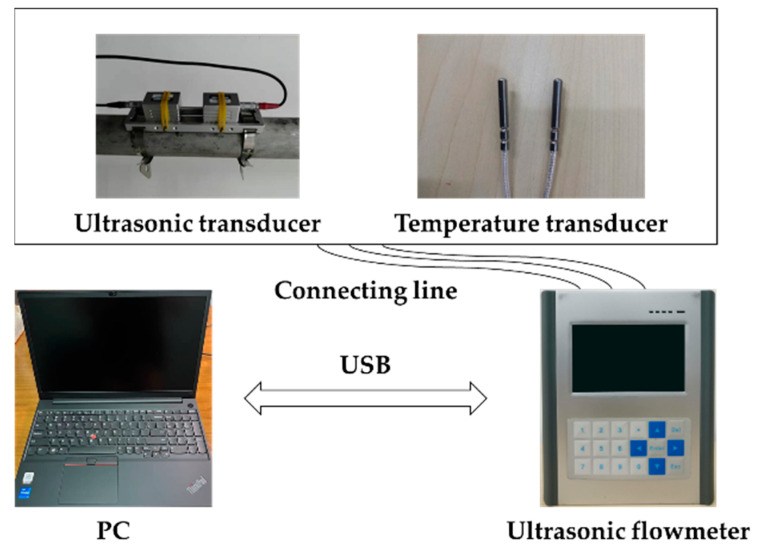
System composition of ultrasonic flowmeter.

The composition of the ultrasonic flowmeter system is shown in Figure 11. The entire measurement system consists of an ultrasonic flowmeter, an ultrasonic transducer, a temperature sensor, a connecting wire, a fixed bracket, a fixture and a computer, etc. The ultrasonic flowmeter is connected with the ultrasonic transducer clamped on the outside of the pipe through a connecting line and is also connected with the temperature sensor clamped outside the pipe to collect the temperature of the pipe in real time, so as to correct the ultrasonic sound speed through the temperature. Ultrasonic flowmeters generally work independently. If connected to the computer through a USB interface, the flowmeter can be remotely controlled by the computer, and the original information recorded by the instrument can be analyzed and displayed in detail through the USB interface. In addition, calibration data such as pipeline parameters, medium parameters and flow measurement configuration information can also be imported.

### 4.2. Hardware Circuit Design

**Figure 12:**
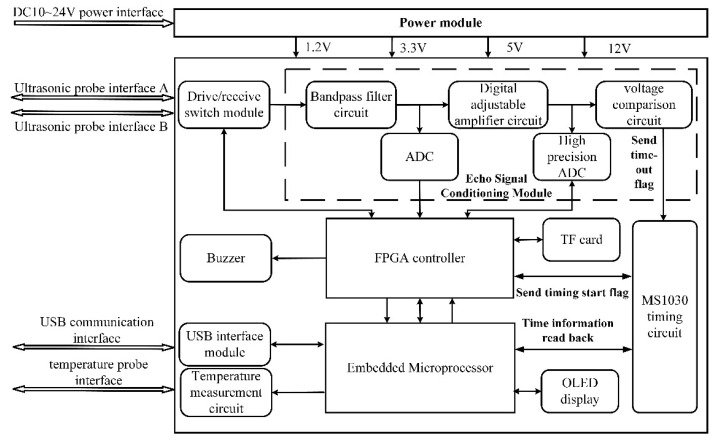
Block diagram of the hardware composition of the ultrasonic flowmeter.

The hardware block diagram of the ultrasonic flowmeter is shown in Figure 12. The hardware system of the ultrasonic flowmeter mainly includes a control module, sensor drive module, echo signal conditioning module, time measurement module, power module and a communication, display and storage module. The control module is mainly composed of an embedded microprocessor and FPGA controller to complete the overall control function of the measurement system. The sensor drive module is mainly used to switch and drive the two ultrasonic transducers and to control the temperature sensor. The echo signal conditioning module mainly completes the filtering, amplification and analogue-to-digital conversion of the signals received by the ultrasonic transducer. The time measurement module mainly completes the time measurement of the ultrasound signal from transmission to reception. The communication, display and storage module is mainly responsible for the ultrasonic flowmeter and the external communication, flowmeter-related parameters and the display of the working status, as well as the storage of information such as echo waveform, velocity and flow.

**Figure 13:**
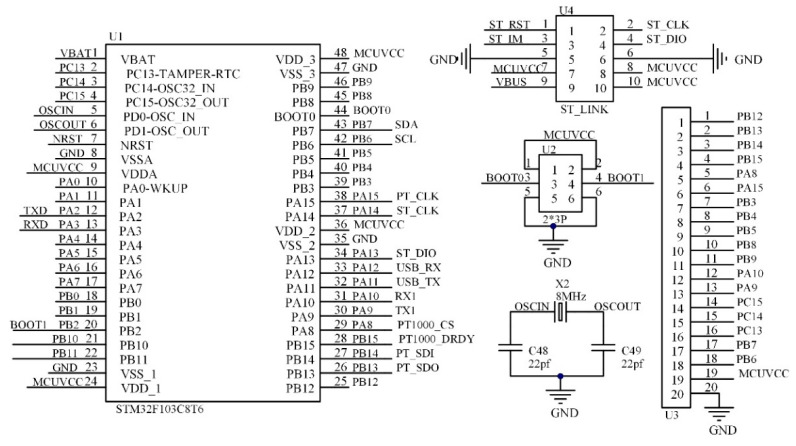
STM32F103C8T6 configuration circuit diagram (2*3P indicates a six-pin row pin.).

The solution proposed in this paper uses a combination of FPGA and an embedded microprocessor for the construction of the ultrasonic flow measurement system hardware, which can play to the strengths of their respective chips and improve the operational efficiency of the system. FPGA has advantages in parallel control and massive data processing, etc., so FPGA is used to control ultrasonic signal transmission, ultrasonic echo signal processing, data storage and time data readback. The embedded microprocessor has advantages in algorithm implementation, so the embedded microprocessor mainly completes the realization of the cross-correlation algorithm and quadratic interpolation algorithm, USB interface communication, temperature measurement, OLED display and time chip control. Among them, the FPGA adopts the XC6SLX9 chip of Xilinx Company, and its maximum operating frequency is 667 MHz; the embedded microprocessor adopts the STM32F103C8T6 developed and produced by STMicroelectronics and uses it as the realization platform of the cross-correlation algorithm. Part of its external configuration circuit is shown in Figure 13. In the figure, U1 is the main processor, U4 is the download interface and U2 is used to select the startup mode of the system. The STM32 is connected to an external crystal oscillator of 8 MHz, and its maximum operating frequency can reach 72 MHz.

**Figure 14:**
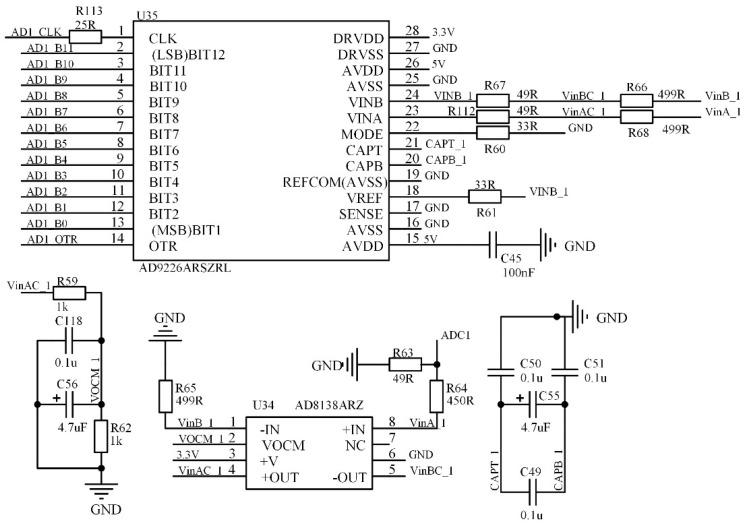
Design of high-precision ADC circuit.

In order to implement the cross-correlation algorithm, an accurate analog signal must be acquired. Since the frequency of the transmitted signal of the ultrasonic transducer can reach up to 5 MHz, a sufficiently high sampling rate must be ensured for analog signal acquisition, so as not to distort the received analog signal. The high-precision ADC circuit design is shown in Figure 14. The selected AD8138 is a single-ended-to-differential amplifier, which can convert single-ended signals into differential signals, and AD9226 is a 12-bit ADC with a sampling rate of 65MSPS, which can realize the acquisition of two channels of analog signals, and the two channels of analog signals work independently.

### 4.3. Software Design

The software part of the ultrasonic flowmeter adopts a modular design. The entire software system includes the main control program, device driver program and function module program, etc., and performs block programming for each function module. The main control program is responsible for calling each software module and is the control core of the entire software system. Each sub-module can be roughly divided into two software modules according to their degree of association, which are the system function switching and parameter configuration module and the flow measurement module.

**Figure 15:**
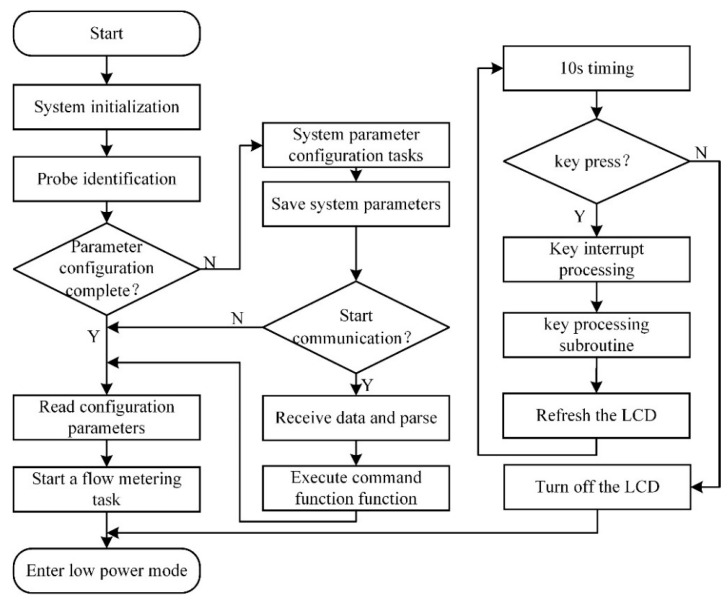
Flow chart of system function switching and parameter configuration module.

The system function switching and parameter configuration module mainly realizes the configuration of the system parameters and measurement parameters. It is used to adjust the calculation results and communicate with the outside world according to different parameters when the flow measurement module is executed. The flow chart is shown in Figure 15. After the system is powered on, it is initialized first, and then the probes with different resonance frequencies are identified. The probes with different resonance frequencies need to be driven by signals of different frequencies. Next, it is judged whether the parameter configuration is completed. If not, the parameter configuration is performed. After the parameter configuration is completed, it is judged whether the parameter configuration is performed through the USB communication interface. If the USB communication interface is activated, the relevant data are received and parsed. The parameter configuration is completed to read the configuration parameters. The configuration parameters mainly include the diameter of the pipe, the material of the pipe, the properties of the fluid and the thickness of the pipe. After reading the configuration parameters, the flow measurement task is started, and the low power consumption mode is entered after the flow measurement is completed. At the same time, the system detects whether the button is pressed in real time through the 10 s timer. If the button is pressed, the interrupt processing is performed, and the LCD is refreshed. If the button is not pressed after 10 s, the LCD is turned off and enters the low power consumption mode.

**Figure 16:**
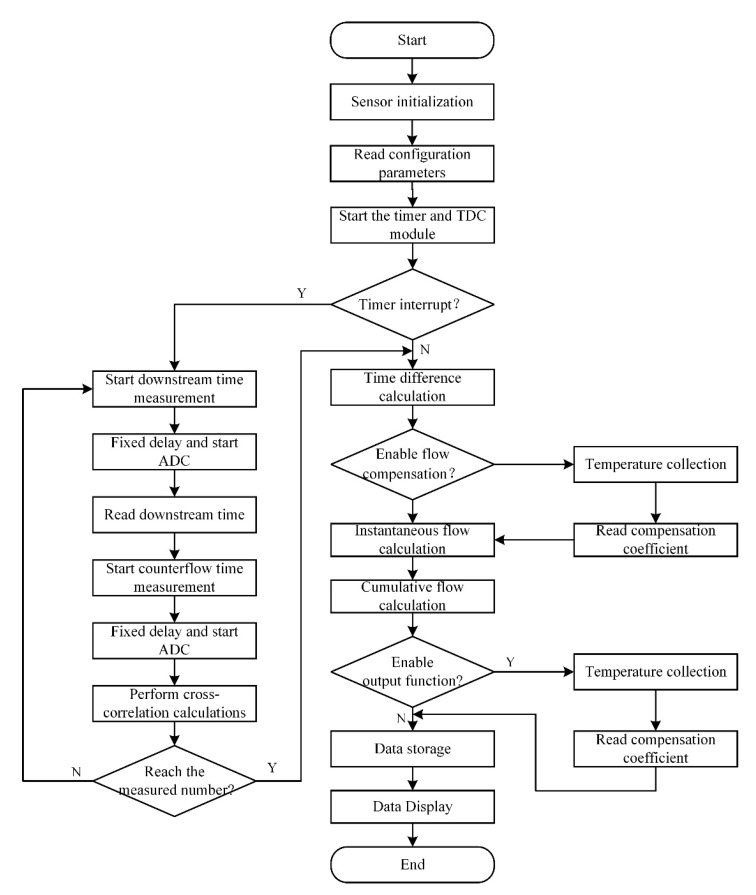
Flow chart of flow measurement module.

The flow chart of the flow measurement module is shown in Figure 16. After the flow measurement task is started, the sensor is initialized first, and then the timing module and the TDC module are started. When the timer interrupt is initiated, the time measurement is started. First, the downstream time measurement is performed, and after completion, the delay is fixed and the ADC is started. Then, the countercurrent time measurement is performed in the same way and the ADC is started. Finally, the cross-correlation calculation is performed. In order to exclude errors caused by a single measurement, multiple measurements are required. If the number of measurements is reached, the time measurement is ended; otherwise, the time measurement is continued, and the results of multiple measurements are averaged. Using the cross-correlation calculation results, the time difference between the upstream and the downstream and the instantaneous flow can be calculated, but there are still some errors in the results. The corrected instantaneous flow is a relatively accurate instantaneous flow. After the flow measurement is completed, the relevant data can be output, stored and displayed.

## 5. System Function Test and Data Analysis

In order to better check the functions and performance indicators of the designed ultrasonic flowmeter, we carried out functional verification and performance testing of the flowmeter on the National Institute of Metrology and the self-built test platform, using the still water dragging method, the dynamic volume method and the field comparison method, respectively.

### 5.1. Still Water Dragging Method

The basic principle of the still water dragging method is that the device being tested is suspended and fixed on the trailer, and the trailer carries the device being tested on the guide rail of the sink to move in a straight line. Assuming that the medium of the sink is completely stationary, the moving speed of the trailer is taken as the standard value of the velocity test to detect the device. The diameter of the pipe being tested t is DN200. During the test, the ultrasonic flowmeter is suspended below the trailer, and the error of its indication value is measured within the speed range of 0.3–1.5 m/s. Each point is measured six times, and its average value and repeatability are counted. The measurement results are shown in Table 2. The test results show that under the condition of DN200 pipe installation, the mean value of the relative indicating error of the flowmeter is less than 0.31%, and the repeatability is less than 0.11%.

### 5.2. Dynamic Volume Method

The basic principle of the dynamic volume method is to measure the falling speed of the liquid level in the container through a high-precision liquid level sensor and multiply the measurement result by the cross-sectional area to obtain a standard flow, so as to detect the measurement performance of the flowmeter. Using the dynamic volume method, the measurement performance of the flowmeter can be evaluated under the conditions of small bore (DN6) and regular bore (DN15, DN26 and DN55).

**Figure 17:**
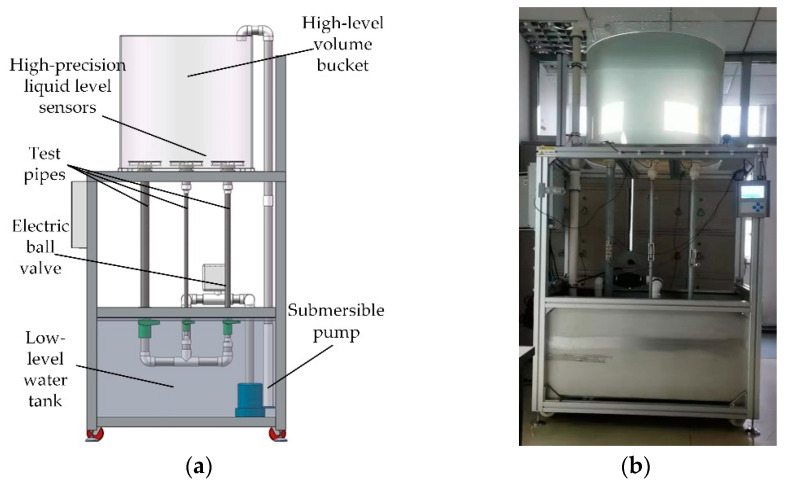
(**a**) Structure diagram of ultrasonic flowmeter test system; (**b**) Physical diagram of ultrasonic flowmeter test system.

The ultrasonic flowmeter measurement system based on the dynamic volume method is mainly composed of a high-level volume bucket, four high-precision liquid level sensors, a low-level water tank, a submersible pump, an electric ball valve and flow test pipes with different pipe diameters. The structure of the ultrasonic flowmeter test system is shown in Figure 17a, and the real object of the test system is shown in Figure 17b. The device can measure the flow of different pipe diameters, and the pipes are all made of PVC. Four liquid level sensors are placed under the high-volume bucket with an inner diameter of 784 mm, a resolution of 1μm, an accuracy of 10 μm and a frequency of 1 MHz, which are used to detect the speed of water falling in the bucket in real time. During the test, the water is pumped from the low-level water tank to the high-level volume bucket by the submersible pump, and the fluid flowing through the pipe is adjusted by opening the electric ball valve to different opening degrees. At this time, the water level falling speed in the volume bucket can be multiplied by the bottom area of the bucket to obtain the standard instantaneous flow, and then the indication error can be calculated by comparing the flow measured by the flowmeter with it.

Under the installation conditions of small-diameter (DN6) pipelines, taking into account the avoidance of the transition zone from laminar flow to turbulent flow, in order to avoid the theoretical uncertainty of the flow velocity distribution coefficient, five flow velocity points in the range of 1.9~2.9 m/s are selected during the detection of the flowmeter, and the test results are shown in Table 3. It can be seen from the measurement results that the absolute value of the relative indication error is not greater than 0.51%, and the flowmeter has the ability to measure flow under the condition of DN6 pipe diameter.

Under the condition of conventional pipe diameter (DN15, DN26 and DN55), the average value of standard flow and flowmeter are measured when the ball valve opening is 30%, 40%, 50%, 60%, 70%, 80%, 90% and 100%, respectively, and the relative indication error is obtained by calculation. The measurement results of DN15, DN26 and DN55 pipe diameter installation conditions are shown in Table 4, Table 5 and Table 6, respectively. It can be seen from the measurement results that, under the condition of DN15 pipe diameter, the absolute value of the relative indication error under different ball valve openings is not more than 0.676%; under the condition of DN26 pipe diameter, the absolute value of the relative indication error under different ball valve openings is not more than 0.597%; and under the condition of DN55 pipe diameter, the absolute value of the relative indication error under different ball valve openings is not more than 0.638%. The flowmeter has the ability to measure flow in DN15, DN26 and DN55 pipe diameters.

### 5.3. Field Comparison Method

In the field comparison method, a multi-channel ultrasonic flowmeter was used as the reference standard. The measurement results of the designed flowmeter were compared with those of the reference flowmeter, and the measurement performance of the flowmeter was evaluated under the conditions of high flow velocity (greater than 20 m/s) and large diameter (DN1600).

When the flow velocity of the fluid medium in the pipeline is high, the existence of cavitation bubbles causes the amplitude beat of the ultrasonic wave and the signal-to-noise ratio to be greatly reduced. When the flow velocity is below 20 m/s, there is no cavitation in the pipeline, and the flow data are relatively stable; when the flow velocity exceeds 20 m/s, the on-site flow noise increases significantly, and the flow fluctuation degree increases. Under the installation condition of DN45 pipe, the stable section is selected for testing at high flow velocity, and the measurement results are shown in Table 7. The data show that the device being tested has flow measurement capability at a high flow velocity of 26 m/s, and the corresponding standard flow is 157 m^3^/h.

Under the installation condition of a large-diameter (DN1600) pipe, five flow velocity points are selected in the range of 0.8~1.6 m/s to test the flowmeter. The test results are shown in Table 8. It can be seen from the measurement results that, under the installation condition of DN1600 pipe diameter, the absolute value of the relative indication error under different ball valve openings is not greater than 0.815%, and the repeatability is not greater than 0.150%. The flowmeter has the capacity of flow measurement in DN1600 pipe diameter.

It can be seen from the above test data that the absolute value of the relative indication error of the ultrasonic flowmeter based on the cross-correlation method designed in this paper is not greater than 0.815%, and the repeatability is not greater than 0.150%. In addition, it has flow measurement capability when the fluid velocity is 26 m/s and the pipe diameter range is DN6~DN1600. The Sitrans FS230 ultrasonic flowmeter, manufactured by Siemens, headquartered in Berlin and Munich, Germany, is also a clamp-on ultrasonic flowmeter and can be used in many fields where liquid flow measurement is required. By consulting the relevant manuals, it can be seen that the measurement accuracy of this flowmeter is ±0.5~1%, and the repeatability is ±0.25%. It can be applied to pipelines with an upper limit of liquid velocity of ±12 m/s and a pipe diameter range of 12.7 mm to 10 m. Compared with the test performance of the Sitrans FS230 ultrasonic flowmeter, the ultrasonic flowmeter designed in this paper has higher measurement accuracy, better repeatability, a higher applicable upper-limit fluid flow rate and can be applied to smaller pipe diameters. However, the measured data are preliminary functional verification and performance test results and have not undergone accurate index testing by a professional metrology agency. Further research can be continued to better refine its performance.

## 6. Conclusions

Compared with traditional flow measurement technology, ultrasonic flow measurement technology has obvious advantages in measurement accuracy, measurement range and portability. In this paper, according to the actual demand of ultrasonic flow measurement, combined with the research status and development trend of the same type of ultrasonic flowmeter at home and abroad, the relevant theory of ultrasonic flow measurement is studied, and the design of the clamp-on ultrasonic flowmeter is proposed based on the cross-correlation method.

In this paper, the modeling, simulation and analysis of ultrasonic flow measurement are carried out through COMSOL software, and the background flow under different fluid states and the influence of pipe diameter on the propagation of ultrasonic signals are analyzed. The implementation process of the cross-correlation algorithm is simulated through Python, which lays the foundation for the subsequent application of the cross-correlation algorithm. The designed combination of embedded microprocessor and FPGA can give full play to their respective advantages and improve the operating efficiency of the system.

In order to verify the feasibility of the design scheme, we used the still water dragging method, the dynamic volume method and the field comparison method, respectively, to carry out functional verification and performance test on the Chinese Academy of Sciences and the self-built test platform. The test results show that the absolute value of the relative indication error of the flowmeter under the conditions of small diameter (DN6), conventional diameter (DN15, DN26 and DN55) and large diameter (DN1600) is not more than 0.815%, and the repeatability is not more than 0.150%, and also has the ability to measure flow at high flow velocity (26 m/s). The measured flow is as low as 0.088 m^3^/h and as high as 157.000 m^3^/h. The designed ultrasonic flowmeter has high precision, good repeatability, strong stability and a wide application range, and can be used in agricultural production, industrial manufacturing, national defense construction and other fields.

## Figures and Tables

**Table 1:** Comparison of different interpolation fitting errors.

Theoretical Time Difference (µs)	Actual Time Difference (µs)	Actual Error (%)	Quadratic Interpolation Error (%)	Cubic Spline Interpolation Error (%)
0.160	0.200	24.999	−2.531	−2.239
0.170	0.200	17.647	0.820	0.974
0.180	0.200	11.111	−0.360	−0.178
0.190	0.200	5.263	0.257	0.478
0.200	0.200	0.000	1.118	1.269
0.210	0.200	−4.761	−1.926	−1.833
0.220	0.200	−9.091	0.207	0.267
0.230	0.200	−13.040	−0.467	−0.430
0.240	0.200	−16.670	0.684	0.768
0.250	0.200	−20.000	−0.698	−0.695

**Table 2:** Still water dragging method test data (DN200).

Flow Velocity Point (mm/s)	Average Value of the Standard Flow Velocity (mm/s)	Average Value of the Measured Flow Velocity (mm/s)	Standard Deviation (%)	Number of Samples	Average Value of Relative Indication Error (%)	Repeatability (%)
300	299.60	299.75	0.34	74	0.05	0.11
400	399.46	399.33	0.29	54	−0.03	0.05
499	499.34	499.08	0.23	41	−0.05	0.04
599	599.21	599.36	0.23	33	0.03	0.09
700	699.81	699.64	0.21	28	−0.02	0.05
800	799.69	800.00	0.19	24	0.04	0.05
900	899.58	899.20	0.20	20	−0.04	0.03
1000	999.43	999.20	0.19	17	−0.02	0.06
1099	1099.33	1099.28	0.16	15	−0.01	0.07
1199	1199.18	1199.03	0.20	13	−0.01	0.05
1300	1299.77	1298.18	0.23	12	−0.12	0.06
1400	1399.63	1397.13	0.23	10	−0.18	0.06
1500	1499.46	1494.76	0.24	9	−0.31	0.05

**Table 3:** Measurement results under DN6 pipe diameter.

Flow Point (m/s)	Average of the Change in Level Height per Second (mm)	Standard Flow (m^3^/h)	Measured Flow (m^3^/h)	Relative Indication Error (%)
1.936	0.050	0.088	0.088	0.11
2.155	0.056	0.098	0.097	−0.10
2.389	0.062	0.108	0.108	−0.28
2.611	0.068	0.118	0.118	−0.51
2.822	0.074	0.128	0.127	−0.31

**Table 4:** Measurement results under DN15 pipe diameter.

Ball Valve Opening (%)	Average of the Change in Level Height per Second (mm)	Average Value of the Standard Flow (m^3^/h)	Average Value of the Measured Flow (m^3^/h)	Relative Indication Error (%)
30	0.187	0.323	0.325	0.464
40	0.487	0.847	0.846	−0.071
50	0.739	1.286	1.283	−0.191
60	0.909	1.585	1.579	−0.379
70	1.006	1.747	1.747	−0.017
80	1.060	1.830	1.842	0.676
90	1.075	1.871	1.867	−0.186
100	1.079	1.883	1.875	−0.427

**Table 5:** Measurement results under DN26 pipe diameter.

Ball Valve Opening (%)	Average of the Change in Level Height per Second (mm)	Average Value of the Standard Flow (m^3^/h)	Average Value of the Measured Flow (m^3^/h)	Relative Indication Error (%)
30	0.296	0.516	0.514	−0.314
40	0.543	0.937	0.943	0.589
50	0.933	1.625	1.620	−0.343
60	1.325	2.308	2.301	−0.294
70	1.665	2.876	2.893	0.597
80	1.868	3.256	3.244	−0.370
90	2.002	3.460	3.478	0.524
100	2.060	3.568	3.579	0.299

**Table 6:** Measurement results under DN55 pipe diameter.

Ball Valve Opening (%)	Average of the Change in Level Height per Second (mm)	Average Value of the Standard Flow (m^3^/h)	Average Value of the Measured Flow (m^3^/h)	Relative Indication Error (%)
30	0.552	0.956	0.958	0.195
40	0.740	1.280	1.285	0.365
50	0.998	1.737	1.733	−0.287
60	1.744	3.044	3.029	−0.493
70	2.507	4.341	4.354	0.294
80	3.727	6.443	6.473	0.462
90	4.540	7.936	7.886	−0.638
100	5.370	9.291	9.328	0.398

**Table 7:** Measurement results at high flow velocity.

Standard Flow (m^3^/h)	Standard Flow Velocity (m/s)	Measured Flow Velocity (m/s)	Average Value of the Measured Flow Velocity (m/s)	Average Value of Relative Indication Error (%)
157.000	26.331	25.930	26.303	−0.108
		26.675		
131.200	22.008	21.882	21.952	−0.250
		22.024		

**Table 8:** Measurement results under DN1600 pipe diameter.

Flow Velocity Point (m/s)	Standard Flow (m^3^/h)	Measured Flow (m^3^/h)	Relative Indication Error of a Single Point (%)	Average value of Relative Indication Error (%)	Repeat-ability (%)
1.51	10947.600	10875.600	−0.658	−0.615	0.037
	10929.600	10864.800	−0.593		
	10886.400	10821.600	−0.595		
1.34	9694.800	9640.800	−0.557	−0.643	0.150
	9712.800	9633.600	−0.815		
	9720.000	9666.000	−0.556		
1.18	8528.400	8478.000	−0.591	−0.548	0.043
	8542.800	8499.600	−0.506		
	8546.400	8499.600	−0.548		
0.98	7059.600	7012.800	−0.663	−0.645	0.078
	7074.000	7023.600	−0.712		
	7066.800	7027.200	−0.560		
0.81	5868.000	5828.400	−0.675	−0.695	0.094
	5871.600	5824.800	−0.797		
	5878.800	5842.800	−0.612		

## Data Availability

Not applicable.
